# Supplementary material for: Spleen tyrosine kinase mediates innate and adaptive immune crosstalk in SARS‐CoV‐2 mRNA vaccination
Source: EMBO Mol Med. 2022 Jul 4;14(8):e15888. doi: 10.15252/emmm.202215888 (PMC9349614; doi:10.15252/emmm.202215888)
Supplement: Supplementary file 1 — Expanded View Figures PDF [file EMMM-14-e15888-s002.pdf]

Expanded View Figures

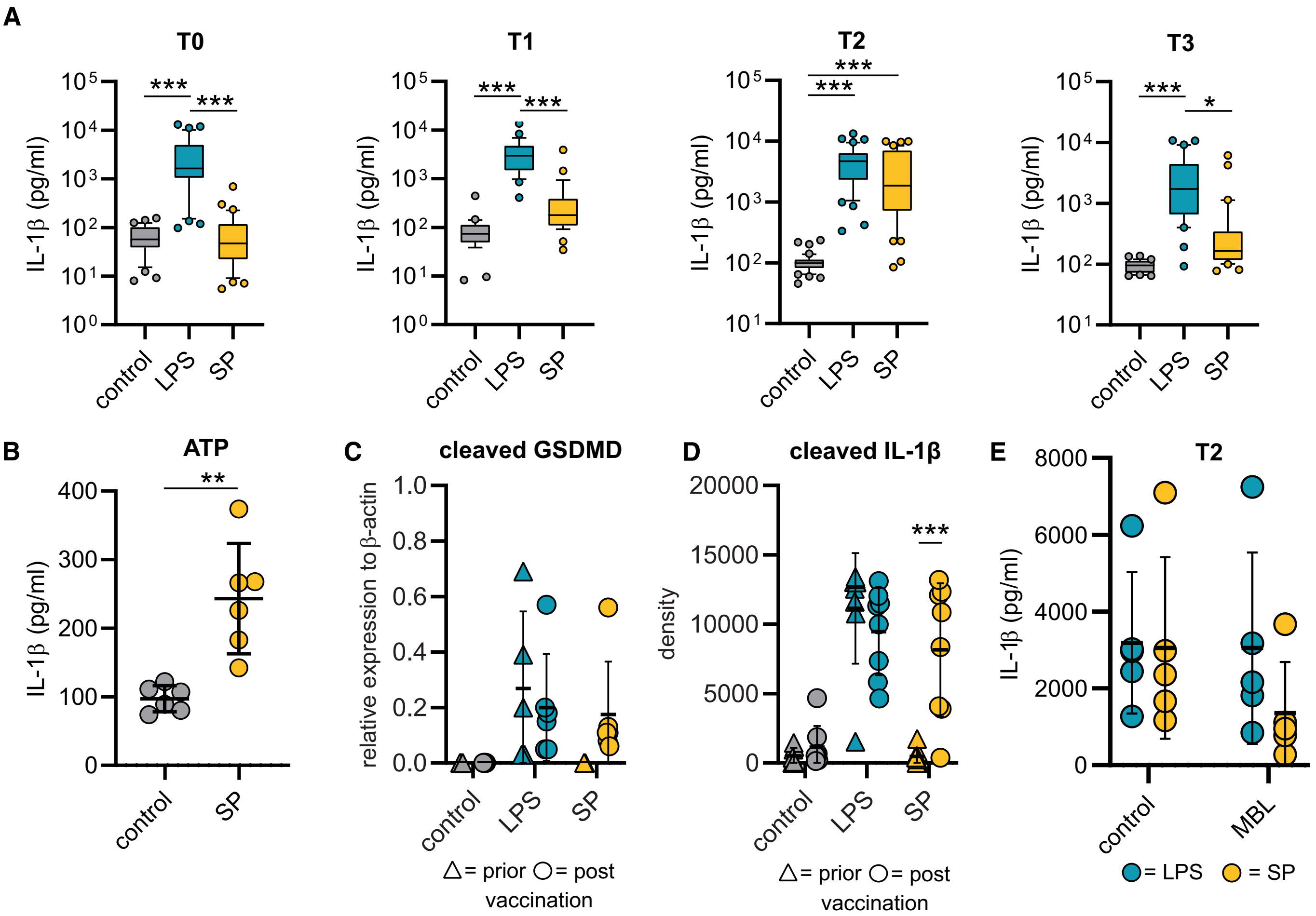

Figure EV1. NLRP3 inflammasome activation in macrophages.

A Monocytes were isolated prior (T0; *n* = 35 individuals) or after vaccination [2 weeks after 1st (T1; *n* = 28 individuals), 2 (T2; *n* = 44 individuals) and 10 (T3; *n* = 31 individuals) weeks after 2nd vaccination] and cultivated in presence of M-CSF for macrophage differentiation. After 5 days IL-1β concentrations were determined by ELISA in supernatants of macrophages, which were left unstimulated (control) or stimulated with the SP/N or LPS/N for 4 h.

B IL-1β concentrations (pg/ml) in macrophages (*n* = 6 individuals), which were left unstimulated or stimulated with the SP following by 2 h incubation with ATP (5 mM).

C, D Quantification of cleaved gasdermin D (cell lysates) (C) and cleaved IL-1β (cell culture supernatants) (D) detected by Western blot in lysate or supernatant of macrophages stimulated with LPS/N or SP/N prior (gasdermin D *n* = 5 individuals; cleaved IL-1β *n* = 8 individuals) and post (gasdermin D *n* = individuals 6; cleaved IL-1β *n* = individuals 8) vaccination. For gasdermin D the relative expression was normalized to β-actin and for cleaved IL-1β absolute values are shown.

E SP/N or LPS/N were incubated with affinity purified mannose-binding lectin (MBL) for 1 h and then added to primary macrophages (*n* = 5). Subsequently, IL-1β was quantified in the supernatants.

Data information: For statistical analysis, one or two-way ANOVA with Tukey *post-hoc* test was used. Box plots indicate the median and the upper and lower quartile. Outliers are plotted as individual dots (outside the 10–90 percentile). Scatter dot plots show mean ± SD. **P* < 0.05; ***P* < 0.01, ****P* < 0.001. (S-protein: SP, lipopolysaccharide: LPS, nigericin: N).

© 2022 The Authors.

Figure EV2. SYK mediated inflammasome activation and cell death.

A Representative immunofluorescence microscopy image of total SYK in primary macrophages 24 h before (upper image) and 14 d after vaccination (lower image). Nuclei were stained with DAPI. Scale bars indicate 20 μm.
B Examples for quantification of pSYK-positive macrophages by flow cytometry before and 14 d after vaccination. Total SYK was measured as control.
C, D Quantification of Phospho-NF-κB (C) and NLRP3 (D) in cell lysates of macrophages prior (Phospho-NF-κB $n = 5$ individuals; NLRP3 = 5 individuals) and post (Phospho-NF-κB $n = 6$ individuals; NLRP3 = 6 individuals) vaccination stimulated with LPS/N or SP/N by Western blot. Relative expression was normalized to β-actin.
E SYK expression quantified by Western blot in THP-1 SYK-knock out cells (SYK^{Ko}) and THP-1 wild-type (WT) cells (control).
F ASC specks (arrows) were quantified microscopically in THP-1 WT and THP-1 SYK^{Ko} cells after stimulation with SP//N. Unstimulated cells were used as control. Scale bars indicate 10 μm.
G ASC formation quantified by immunofluorescence-microscopy in SP/N-stimulated THP-1 SYKKO or wild-type cells ($n = 6$ independent experiments).
H Exemplary gating strategy to determine cell death of T2 macrophages treated with R406 prior to SP/N stimulation (lower plot) compared to untreated cells (upper plot).
I Primary macrophages (T2) were incubated with the SYK-inhibitors entospletinib (5 μM) ($n = 20$ individuals) and R406 (5 μM ($n = 13$ individuals) for 2 h. Subsequently, cells were stimulated with LPS/N or SP/N. IL-1β concentrations were measured by ELISA. DMSO-treated, LPS-stimulated and untreated unstimulated macrophages were used as control ($n = 43$ individuals).
J Mitochondrial reactive oxygen species (mROS, superoxide) were quantified by flow cytometry and MitoSOX red staining at T2 in SP/N-stimulated untreated macrophages (DMSO; gray) and in cells treated with R406 (orange) before stimulation.
K SOD2 detected by Western Blot in cell lysates of macrophages stimulated with LPS/N or SP/N prior ($n = 5$ individuals) and post ($n = 7$ individuals) vaccination. Relative expression was normalized to β-actin.
L Flow cytometry gating strategy for quantification of changes in mitochondrial membrane potentials: T0 and T2 macrophages were stimulated with SP/N and stained with tetramethylrhodamine methyl ester (TMRM). Unstimulated cells were used as control.
M T2 macrophages were treated with DMSO as control or with R406. Subsequently, cells were stimulated with SP/N and stained with TMRM.
N Primary macrophages from individuals ($n = 5$) after vaccination were treated with MitoTEMPO (10 μM) and stimulated with SP, LPS or left untreated. IL-1β release was quantified after nigericin stimulation.

Data information: For statistical analysis two-way ANOVA with Tukey *post-hoc* test was used. For analysis of Western Blot data, significance was calculated by multiple *t*-test between the expression prior and post vaccination. Box plots indicate the median and the upper and lower quartile. Outliers are plotted as individual dots (outside the 10–90 percentile). Scatter dot plots show mean ± SD. $^*P < 0.05$; $^{**}P < 0.01$, $^{***}P < 0.001$. (S-protein: SP, lipopolysaccharide: LPS, nigericin: N).

© 2022 The Authors.

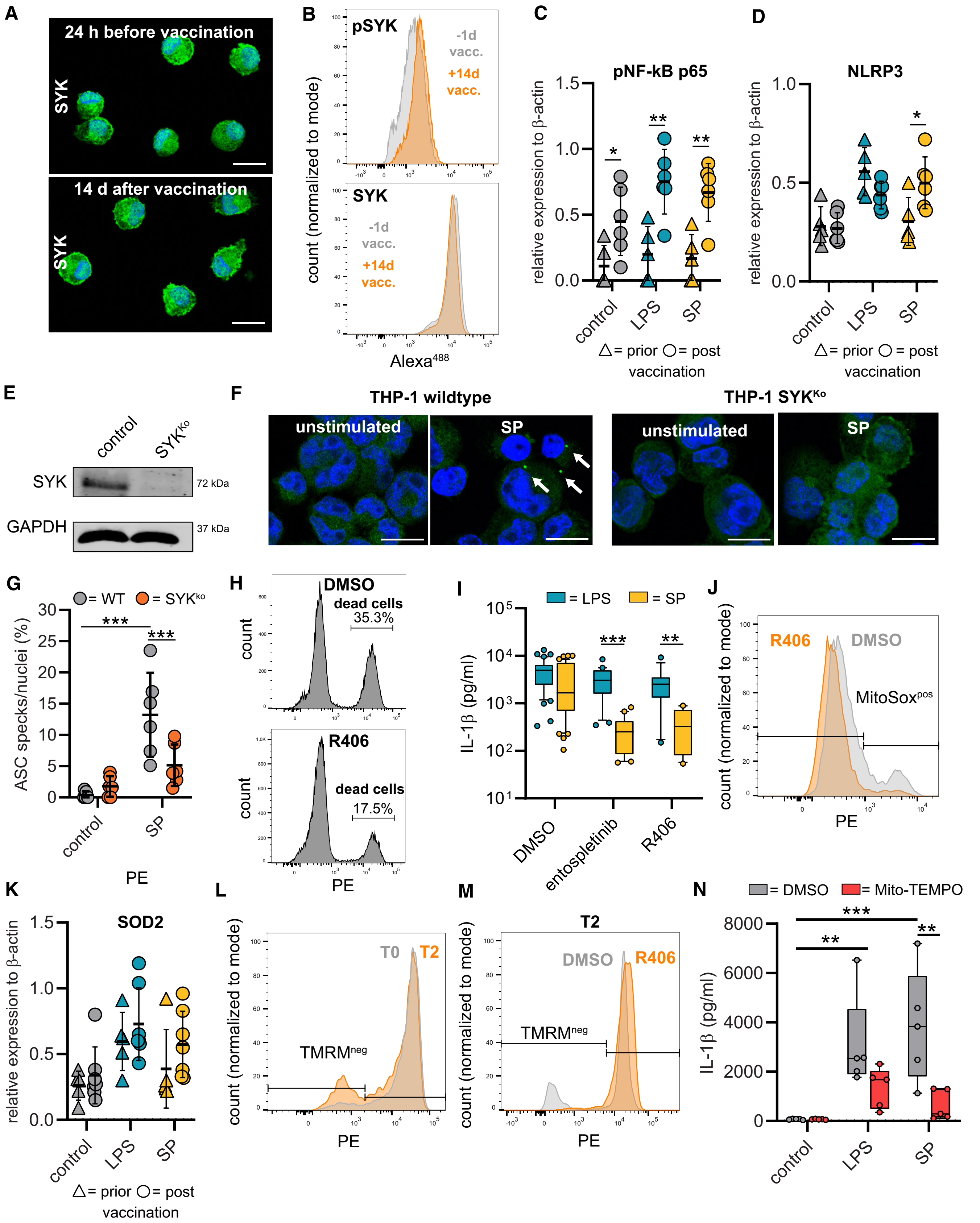

Figure EV2.

 © 2022 The Authors.

Figure EV3. Macrophage-dependent activation of T cells.

A Experimental scheme of co-stimulation experiments: For all experiments, autologous macrophages and T cells were used. Prior to co-culture, monocytes were isolated, differentiated for 5 days and stimulated as described. The day prior to co-culture, total PBMCs were thawed, and T cells were activated for 24 h at 37°C using human IL-2, anti-CD3 and anti-CD28 antibodies. After stimulation, macrophage and T cell cultures were washed and co-cultures were set up in 1:1 ratio in 96-well plate. Cocultures were further incubated at 37°C for 24 h. Subsequently, T cell subpopulations were quantified by flow cytometry.

B Flow cytometry gating strategy of $CD4^+$ and $CD8^+$ T cell subpopulation (N = naïve, CM = central memory, EM = effector memory, TE = terminal effector) of co-cultures with macrophages stimulated with SP/N at T0 and T2.

C, D Fold change of $CD4^+$ terminal effector T cell (n = 11 individuals) (C) and $CD8^+$ terminal effector T cell (n = 11 individuals) (D) populations upon co-culture of macrophages stimulated with SP/N in presence of R406 or MCC950. DMSO was used as control.

E, F PD-1 expression in $CD4^+$ terminal effector T cells (n = 11 individuals) (E) and $CD8^+$ terminal effector T cells (n = 11 individuals) (F) upon co-culture with macrophages stimulated with SP/N in presence of R406 or MCC950. DMSO was used as control.

G, H (G) TNF-α was quantified in the supernatants of the corresponding co-cultures (T0: n = 6 individuals; T2: n = 11 individuals), which were also treated with MCC950 or R406 (H).

Data information: For statistical analysis, one or two-way ANOVA with Tukey *post-hoc* test was used. Graphs show mean ± SD *$P < 0.05$; **$P < 0.01$, ***$P < 0.001$. (S-protein: SP, lipopolysaccharide: LPS, nigericin: N).

© 2022 The Authors.

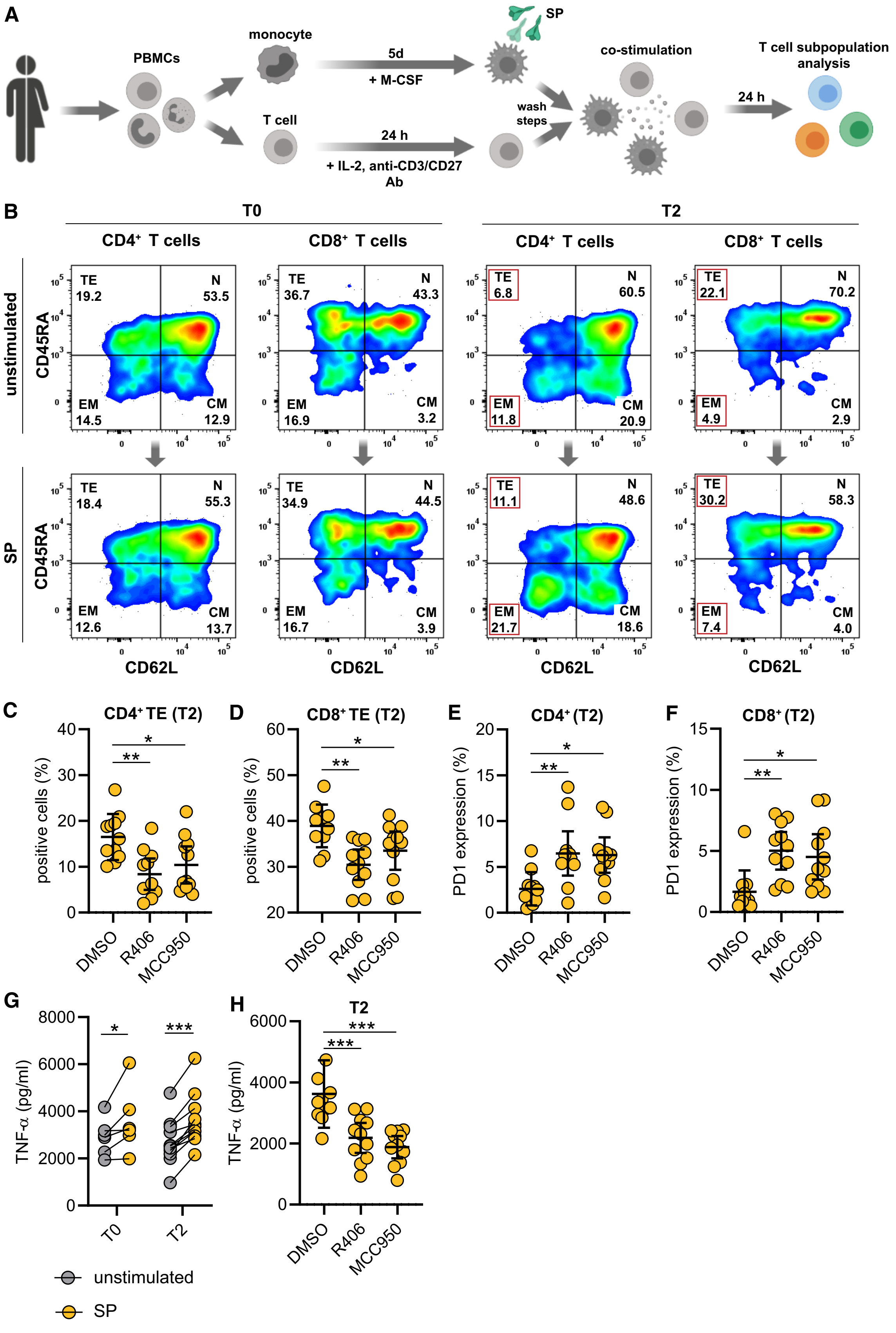

Figure EV3.

© 2022 The Authors.

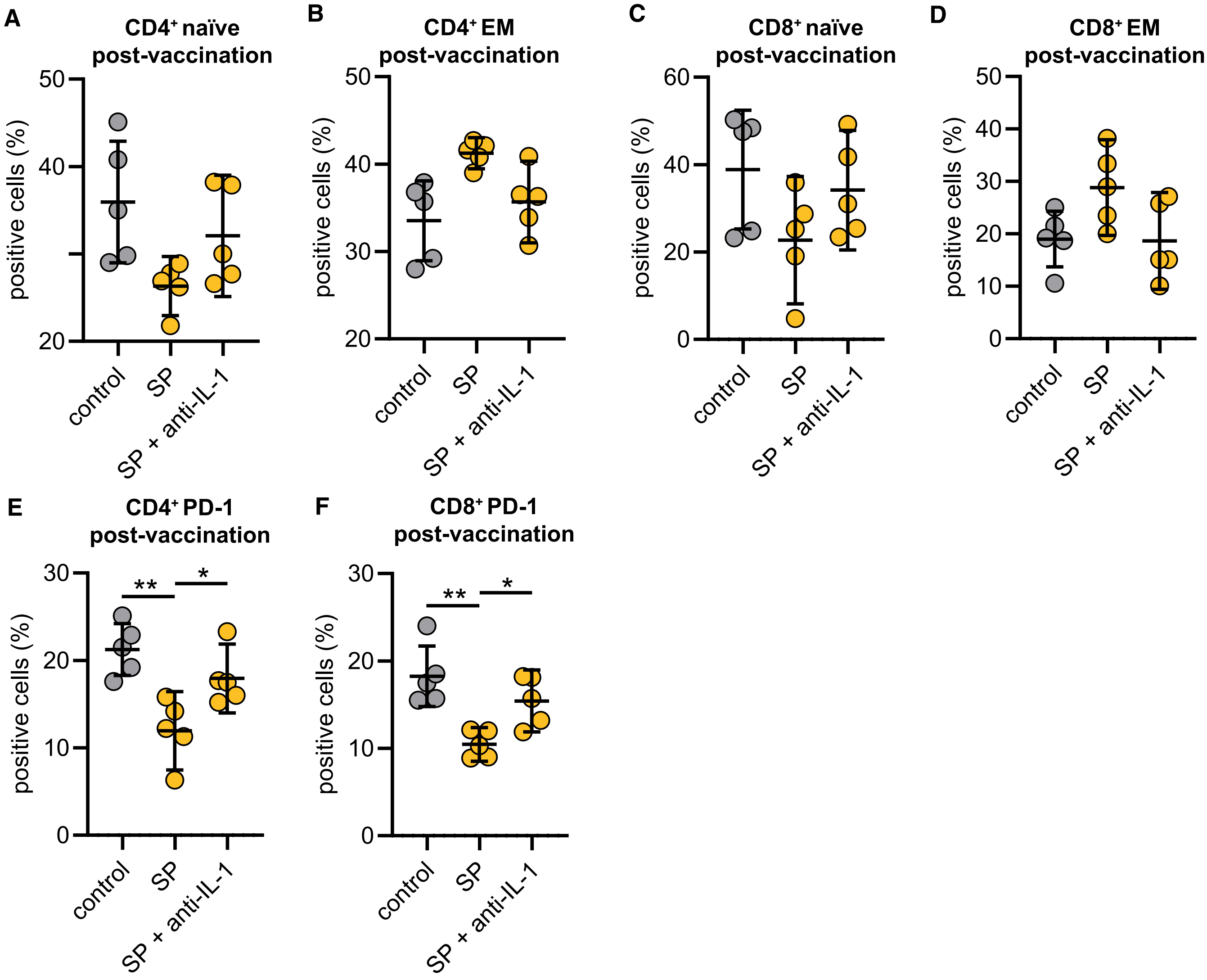

Figure EV4. Macrophage-induced T-cell activation can be abrogated by IL-1β blockade.

A–D Costimulation experiments were conducted as explained in EV3A with or without the presence of an IL-1β specific antibody (1 μg/ml) with cells from individuals' post-vaccination (n = 5). Subsequently, $CD4^+$ naïve (A), effector memory (B), $CD8^+$ naïve, (C) and effector memory (D) populations were quantified by flow cytometry.

E, F PD-1 expression in $CD4^+$ terminal effector T cells (E) and $CD8^+$ terminal effector T cells (F) upon co-culture with macrophages from individuals post-vaccination (n = 5) stimulated with SP/N in presence of the IL-1β specific antibody.

Data information: For statistical analysis, one-way ANOVA was used. Graphs show mean ± SD $*P < 0.05$; $**P < 0.01$. (S-protein: SP, IL-1β specific antibody: anti-IL-1).

© 2022 The Authors.
