# Supplementary material for: Spleen tyrosine kinase mediates innate and adaptive immune crosstalk in SARS‐CoV‐2 mRNA vaccination
Source: EMBO Mol Med. 2022 Jul 4;14(8):e15888. doi: 10.15252/emmm.202215888 (PMC9349614; doi:10.15252/emmm.202215888)
Supplement: Supplementary file 2 — Table EV1 [file EMMM-14-e15888-s001.docx]

**Table EV1. Differentially expressed genes (padj < 0.05 Log2FC > 1):**

Differenitally expressed genes identified in RNA-seq experiments with macrophages from vaccinated vs unvaccinated individuals

| **Gene name** | **Gene ID** | **pvalue** | **padj** | **Log2FC** |
| --- | --- | --- | --- | --- |
| CCL20 | ENSG00000115009 | 1.675E-07 | 9.22E-05 | 6.085645 |
| CCR7 | ENSG00000126353 | 1.077E-07 | 6.32E-05 | 5.441714 |
| HCAR2 | ENSG00000182782 | 6.329E-08 | 4.13E-05 | 5.418387 |
| CCL5 | ENSG00000271503 | 6.284E-07 | 0.000257 | 5.332449 |
| PTGS2 | ENSG00000073756 | 5.544E-07 | 0.000244 | 5.206738 |
| IL1A | ENSG00000115008 | 2.92E-06 | 0.00063 | 4.808943 |
| UNC13A | ENSG00000130477 | 5.999E-07 | 0.000252 | 4.718238 |
| CXCL1 | ENSG00000163739 | 1.047E-05 | 0.001444 | 4.660126 |
| CYP7B1 | ENSG00000172817 | 4.62E-06 | 0.000848 | 4.477125 |
| LINC02605 | ENSG00000261618 | 1.631E-06 | 0.000399 | 4.369706 |
| GBP4 | ENSG00000162654 | 3.666E-06 | 0.000742 | 4.3023 |
| TNIP3 | ENSG00000050730 | 1.871E-06 | 0.000445 | 4.20861 |
| HCAR3 | ENSG00000255398 | 1.207E-06 | 0.000354 | 4.160917 |
| ELOVL7 | ENSG00000164181 | 1.417E-06 | 0.000373 | 4.031816 |
| GCH1 | ENSG00000131979 | 6.837E-06 | 0.001075 | 3.858937 |
| IL23A | ENSG00000110944 | 1.561E-05 | 0.00195 | 3.777571 |
| CCL3L3 | ENSG00000276085 | 1.049E-05 | 0.001444 | 3.760792 |
| CCL4L2 | ENSG00000276070 | 1.378E-05 | 0.001762 | 3.760505 |
| IL1B | ENSG00000125538 | 1.978E-05 | 0.002323 | 3.736537 |
| INHBA | ENSG00000122641 | 1.179E-05 | 0.001561 | 3.722174 |
| CXCL8 | ENSG00000169429 | 5.927E-07 | 0.000252 | 3.705142 |
| AC083837.1 | ENSG00000285744 | 1.467E-05 | 0.00186 | 3.478438 |
| CCL4 | ENSG00000275302 | 3.874E-06 | 0.000767 | 3.433806 |
| LAMP3 | ENSG00000078081 | 2.079E-05 | 0.00241 | 3.414552 |
| ADORA2A | ENSG00000128271 | 4.693E-05 | 0.004374 | 3.356549 |
| TNC | ENSG00000041982 | 1.19E-05 | 0.001564 | 3.316597 |
| CD80 | ENSG00000121594 | 1.651E-05 | 0.002033 | 3.204709 |
| TNFSF15 | ENSG00000181634 | 5.106E-12 | 1.28E-08 | 3.202956 |
| KLF5 | ENSG00000102554 | 5.337E-06 | 0.000922 | 3.038413 |
| TNFRSF4 | ENSG00000186827 | 1.338E-06 | 0.000372 | 2.984258 |
| KCNF1 | ENSG00000162975 | 5.002E-06 | 0.000899 | 2.920558 |
| SGPP2 | ENSG00000163082 | 2.45E-05 | 0.002732 | 2.813849 |
| DUSP8 | ENSG00000184545 | 1.271E-05 | 0.001646 | 2.793476 |
| EBI3 | ENSG00000105246 | 0.0001098 | 0.007646 | 2.778296 |
| BAALC | ENSG00000164929 | 1.249E-06 | 0.000361 | 2.697691 |
| AMZ1 | ENSG00000174945 | 2.126E-05 | 0.002448 | 2.69253 |
| FAM186B | ENSG00000135436 | 8.873E-07 | 0.000295 | 2.679986 |
| TRAF1 | ENSG00000056558 | 3.273E-05 | 0.003391 | 2.606086 |
| AC007336.2 | ENSG00000274508 | 4.99E-07 | 0.000225 | 2.599662 |
| TNFAIP6 | ENSG00000123610 | 0.0001434 | 0.009125 | 2.509368 |
| CDC42EP2 | ENSG00000149798 | 8.994E-06 | 0.001309 | 2.489427 |
| FAM124A | ENSG00000150510 | 5.852E-05 | 0.005189 | 2.460836 |
| MN1 | ENSG00000169184 | 1.589E-05 | 0.001971 | 2.458114 |
| LINC01181 | ENSG00000250929 | 9.908E-06 | 0.001396 | 2.406678 |
| IL15RA | ENSG00000134470 | 9.235E-05 | 0.006922 | 2.38179 |
| ARHGAP23 | ENSG00000275832 | 3.299E-07 | 0.000161 | 2.374017 |
| AC025580.2 | ENSG00000259354 | 2.91E-10 | 4.66E-07 | 2.329903 |
| PTGES | ENSG00000148344 | 9.068E-09 | 7.92E-06 | 2.183117 |
| IRAK2 | ENSG00000134070 | 5.209E-06 | 0.000918 | 2.155339 |
| MT1A | ENSG00000205362 | 5.446E-05 | 0.004894 | 2.08566 |
| AL139246.3 | ENSG00000228037 | 4.741E-05 | 0.004395 | 2.042684 |
| N4BP3 | ENSG00000145911 | 4.995E-06 | 0.000899 | 2.000409 |
| CRIM1 | ENSG00000150938 | 7.343E-07 | 0.000275 | 1.942376 |
| IL15 | ENSG00000164136 | 4.401E-05 | 0.004167 | 1.936195 |
| RGS16 | ENSG00000143333 | 8.995E-08 | 5.46E-05 | 1.917431 |
| TTC39B | ENSG00000155158 | 0.0001281 | 0.00842 | 1.906936 |
| CFB | ENSG00000243649 | 0.0002126 | 0.011414 | 1.897374 |
| LCNL1 | ENSG00000214402 | 0.0001971 | 0.01085 | 1.897047 |
| MEF2B | ENSG00000213999 | 1.116E-06 | 0.000333 | 1.893643 |
| MIR3142HG | ENSG00000253522 | 8.46E-06 | 0.001242 | 1.792026 |
| MCOLN2 | ENSG00000153898 | 0.000158 | 0.009566 | 1.774632 |
| JCAD | ENSG00000165757 | 0.0002147 | 0.011441 | 1.721176 |
| TIFA | ENSG00000145365 | 1.934E-08 | 1.48E-05 | 1.717866 |
| TNF | ENSG00000232810 | 0.0002906 | 0.013787 | 1.6944 |
| HMGN2P46 | ENSG00000179362 | 4.654E-07 | 0.000216 | 1.675342 |
| MAILR | ENSG00000253320 | 3.459E-05 | 0.003522 | 1.655371 |
| COL13A1 | ENSG00000197467 | 7.575E-06 | 0.001164 | 1.639464 |
| CD274 | ENSG00000120217 | 6.311E-05 | 0.005396 | 1.625257 |
| MIR3945HG | ENSG00000251230 | 0.0003213 | 0.014622 | 1.621176 |
| CCL3 | ENSG00000277632 | 0.0002019 | 0.011052 | 1.619931 |
| GP1BA | ENSG00000185245 | 0.0001049 | 0.007525 | 1.610828 |
| COL1A1 | ENSG00000108821 | 0.0002224 | 0.011499 | 1.610085 |
| NR4A2 | ENSG00000153234 | 7.844E-05 | 0.006206 | 1.593519 |
| C15orf48 | ENSG00000166920 | 3.151E-07 | 0.000159 | 1.587686 |
| CLEC4E | ENSG00000166523 | 9.171E-06 | 0.001317 | 1.543956 |
| SOD2 | ENSG00000112096 | 6.628E-05 | 0.005586 | 1.522717 |
| SERPINA1 | ENSG00000197249 | 1.254E-11 | 2.55E-08 | 1.520822 |
| PTGDS | ENSG00000107317 | 0.0003047 | 0.014125 | 1.468267 |
| APOBEC3A | ENSG00000128383 | 0.000519 | 0.019552 | 1.461813 |
| ITGA1 | ENSG00000213949 | 0.0005261 | 0.019675 | 1.426482 |
| AF117829.1 | ENSG00000251136 | 1.774E-05 | 0.002155 | 1.389529 |
| SHISA2 | ENSG00000180730 | 0.0004569 | 0.01809 | 1.364853 |
| CKB | ENSG00000166165 | 0.0005393 | 0.019817 | 1.354156 |
| JAG1 | ENSG00000101384 | 0.0001018 | 0.007438 | 1.3274 |
| AC093583.1 | ENSG00000242048 | 0.0001446 | 0.009131 | 1.288863 |
| AKT3 | ENSG00000117020 | 2.051E-05 | 0.002393 | 1.26906 |
| KYNU | ENSG00000115919 | 9.49E-06 | 0.001348 | 1.248006 |
| AC090617.5 | ENSG00000263050 | 0.0001071 | 0.007607 | 1.206198 |
| FAIM2 | ENSG00000135472 | 0.00054 | 0.019817 | 1.183738 |
| ABTB2 | ENSG00000166016 | 0.0005129 | 0.01947 | 1.179202 |
| H4C14 | ENSG00000270882 | 3.508E-06 | 0.000727 | 1.173854 |
| AREG | ENSG00000109321 | 0.0004398 | 0.017728 | 1.159936 |
| APOL3 | ENSG00000128284 | 0.000266 | 0.013016 | 1.151173 |
| COL4A2-AS2 | ENSG00000224821 | 0.0007933 | 0.025261 | 1.136129 |
| SLC2A1 | ENSG00000117394 | 6.52E-05 | 0.005548 | 1.125607 |
| CPAMD8 | ENSG00000160111 | 5.984E-06 | 0.000976 | 1.116789 |
| TREM1 | ENSG00000124731 | 9.897E-05 | 0.007294 | 1.084011 |
| GCNT4 | ENSG00000176928 | 0.0003974 | 0.016748 | 1.069396 |
| SLC6A16 | ENSG00000063127 | 0.0003733 | 0.016172 | 1.061811 |
| SSPOP | ENSG00000197558 | 0.0002944 | 0.013904 | 1.054936 |
| AL159166.1 | ENSG00000231811 | 0.0004725 | 0.018496 | 1.027529 |
| AC004847.1 | ENSG00000260997 | 0.0004883 | 0.018944 | 1.026282 |
| PMAIP1 | ENSG00000141682 | 0.0007411 | 0.024582 | 1.005124 |
