# Supplementary figures and images for: Spleen tyrosine kinase mediates innate and adaptive immune crosstalk in SARS‐CoV‐2 mRNA vaccination
Source: EMBO Mol Med. 2022 Jul 4;14(8):e15888. doi: 10.15252/emmm.202215888 (PMC9349614; doi:10.15252/emmm.202215888)

# Cleaved IL-1 $\beta$

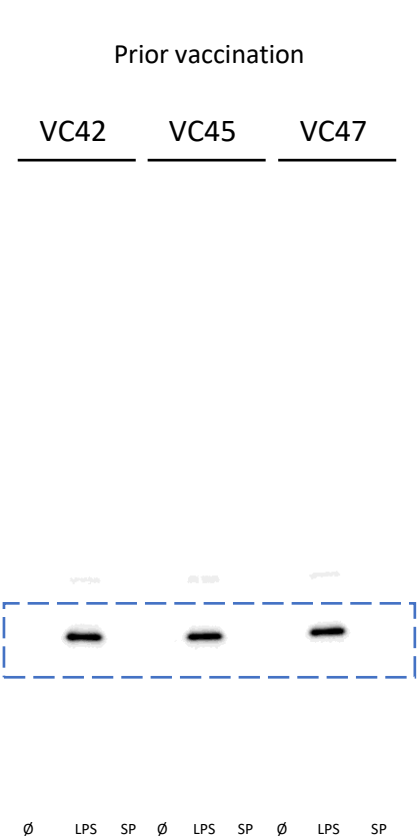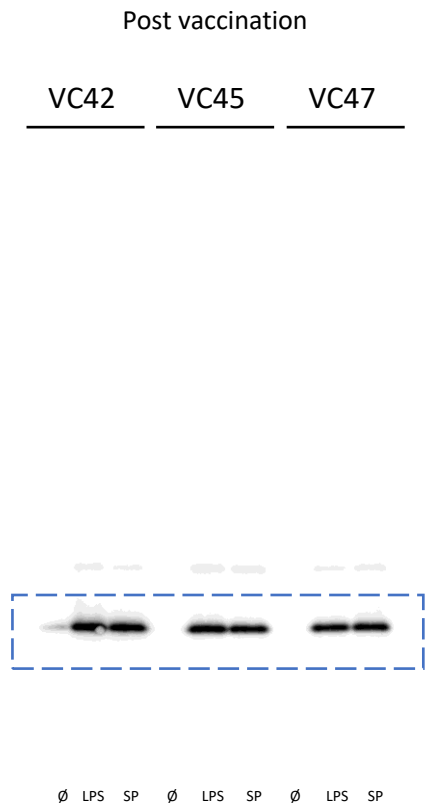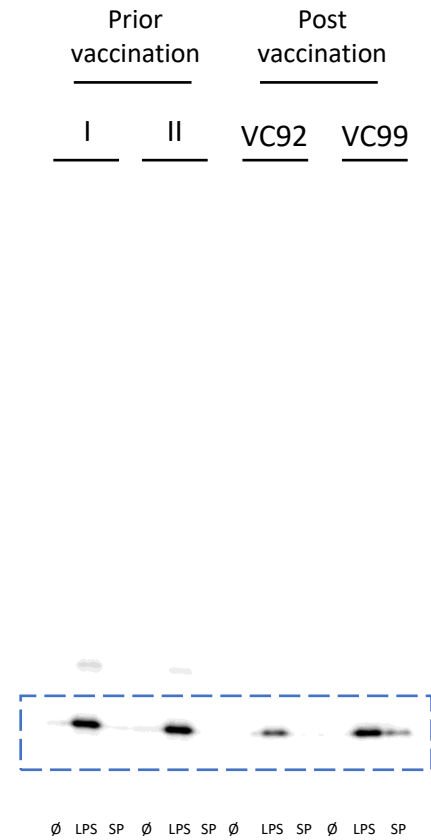

Cleaved IL-1 $\beta$

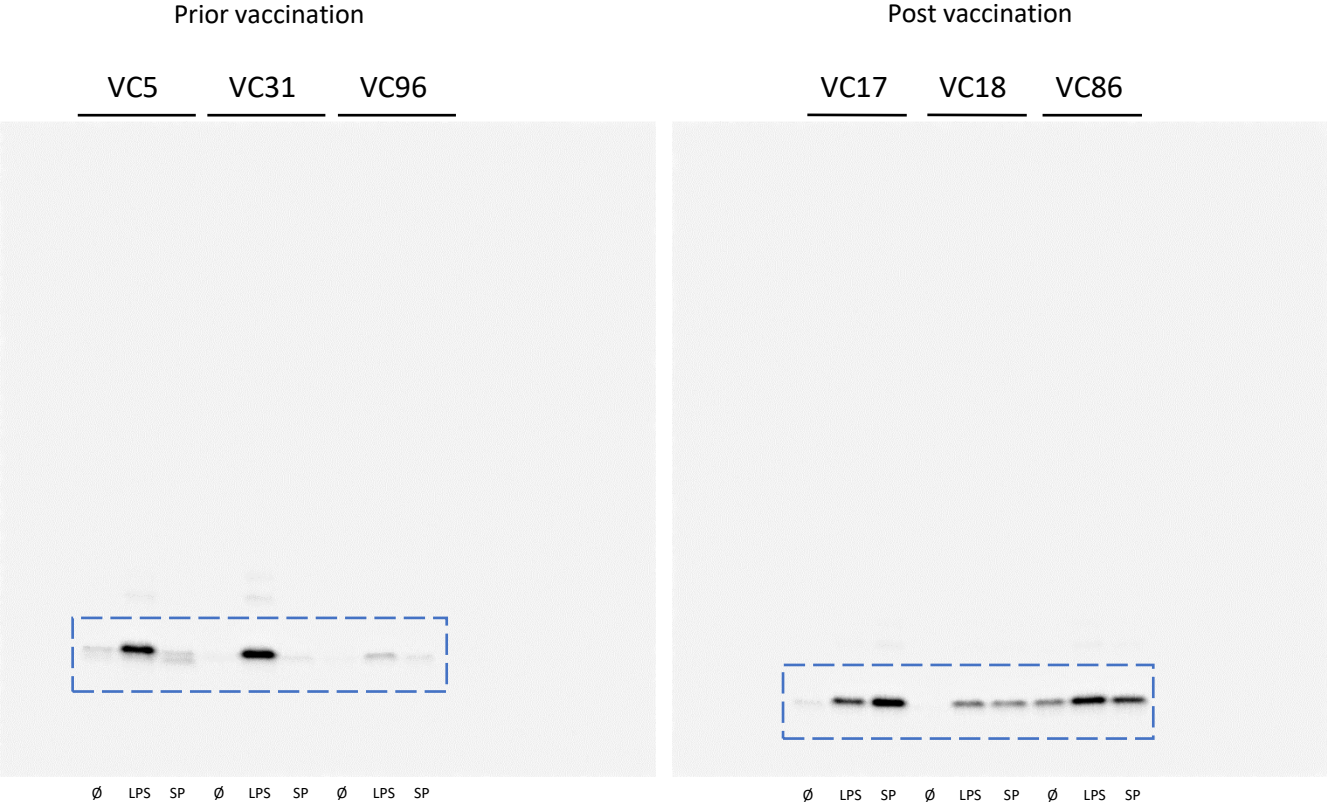

# Cleaved GSDMD

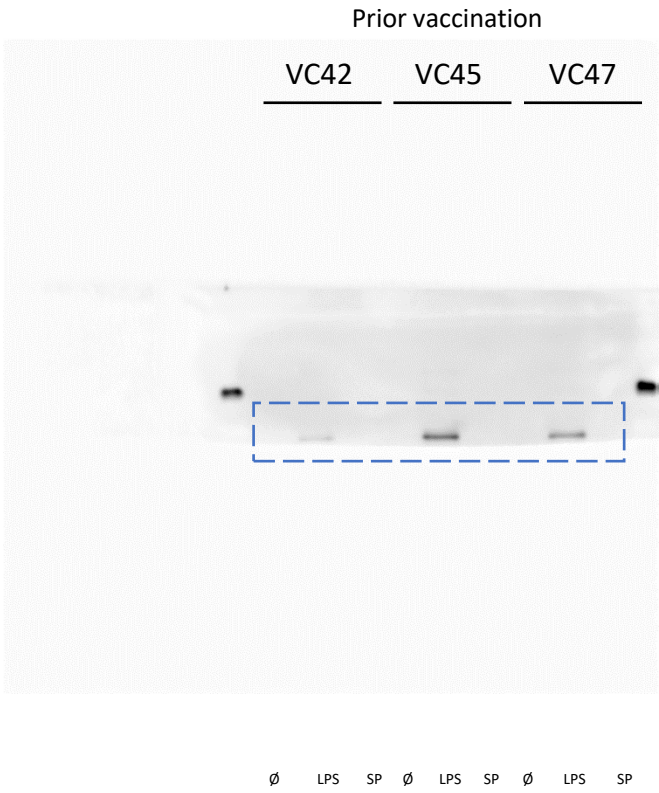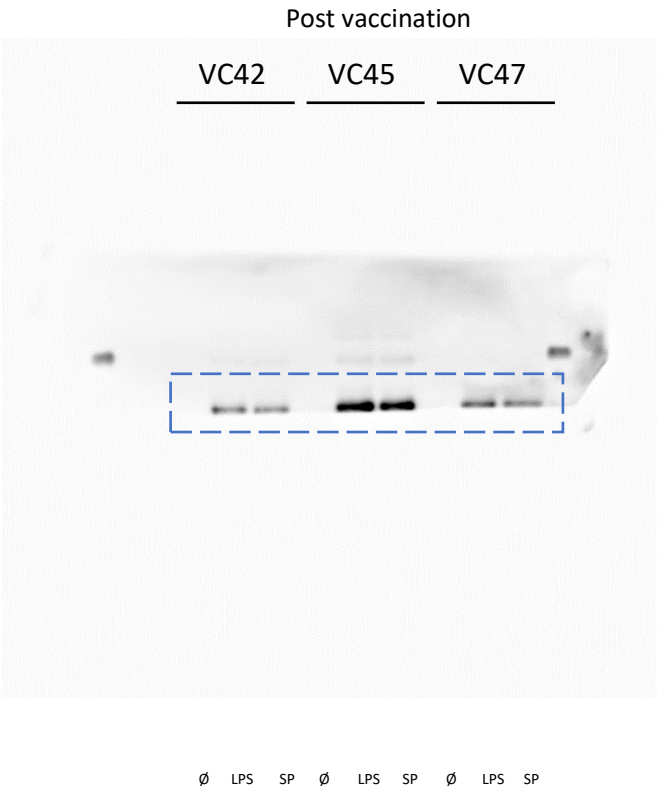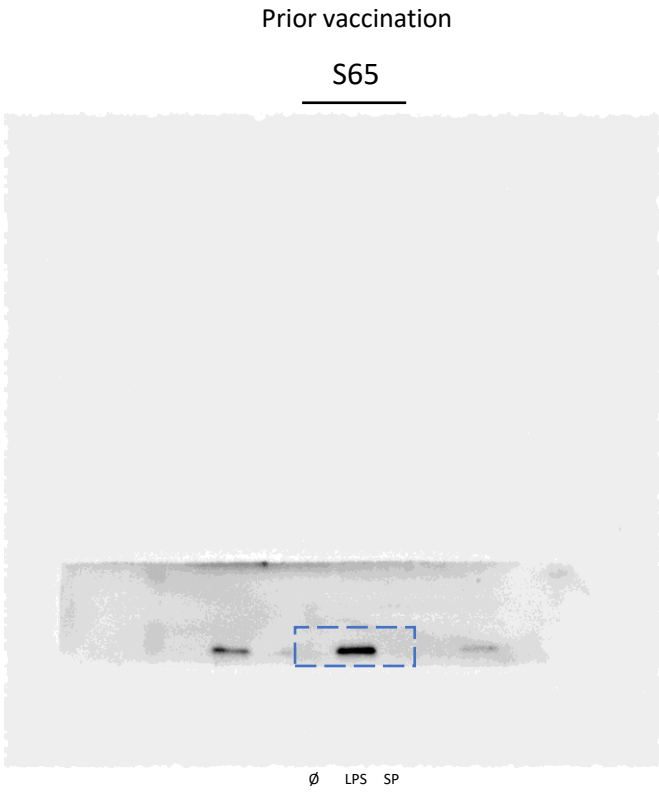

# Cleaved GSDMD

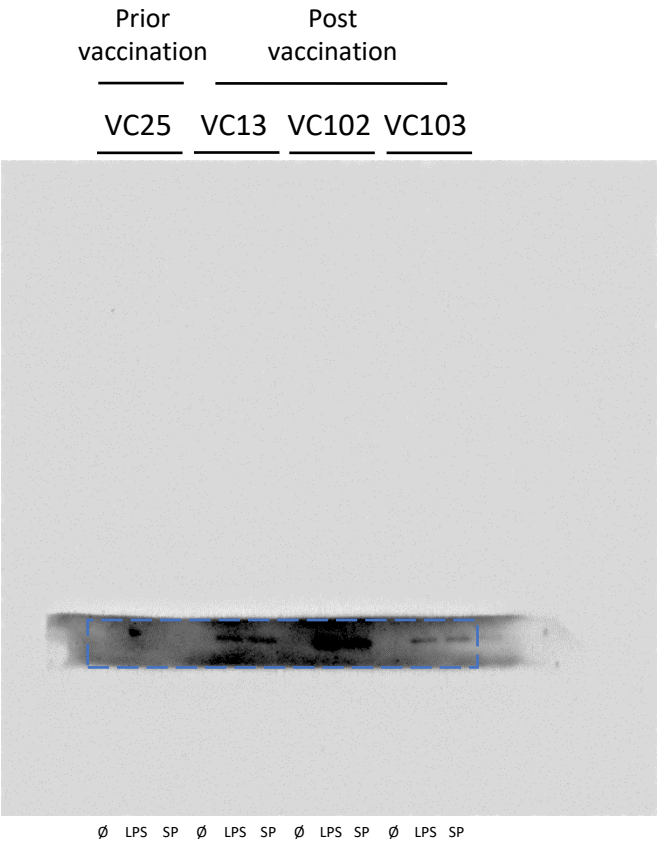

Supplement: Supplementary file 3 — Source Data for Figure 1 [file EMMM-14-e15888-s005.zip › Source data Fig1.pdf]

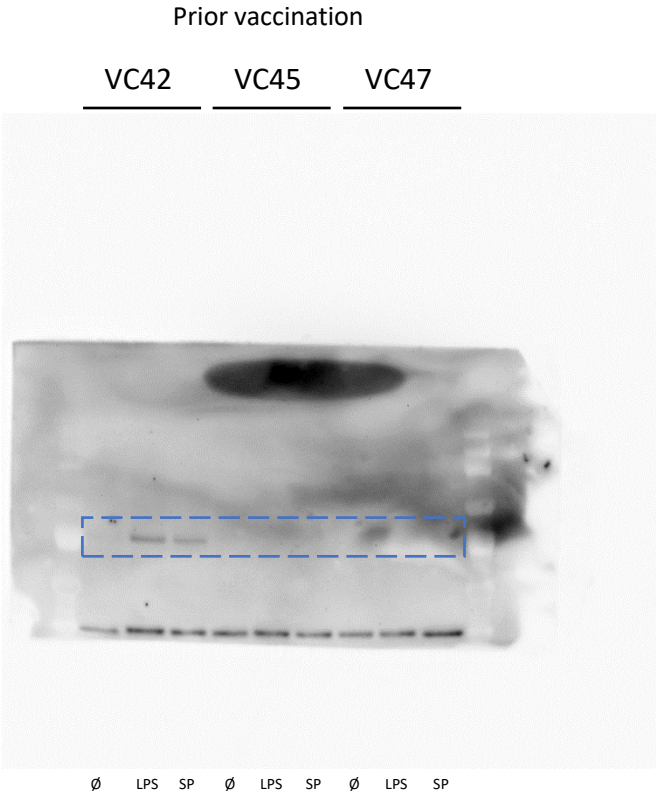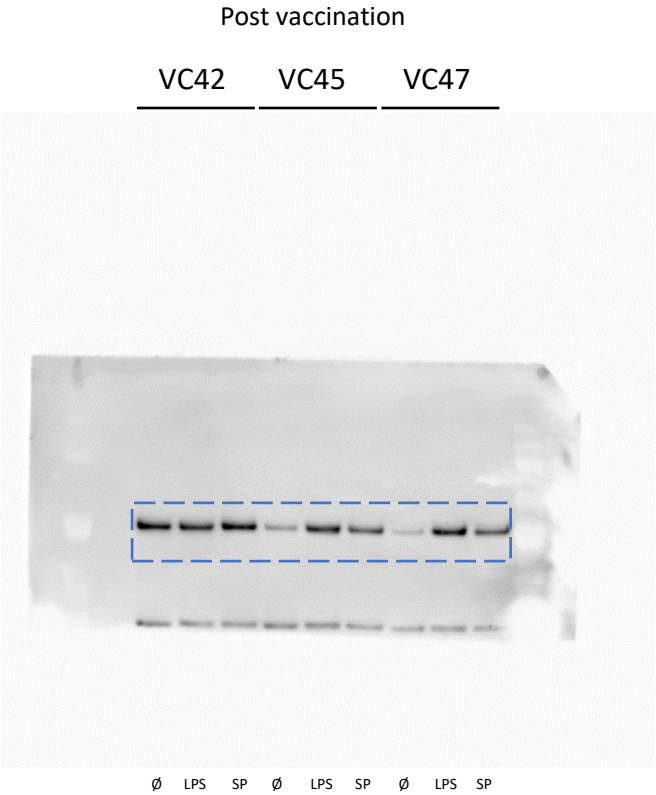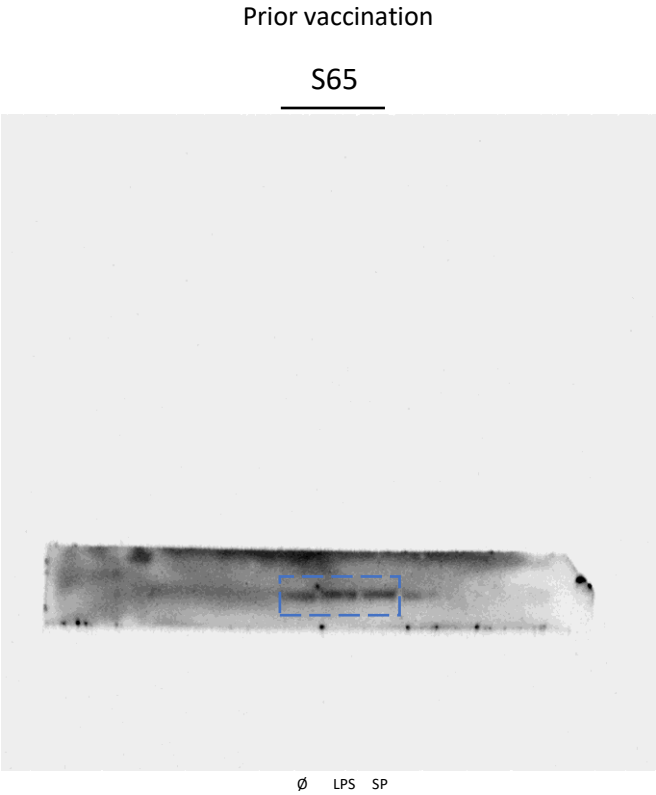

pNFkB

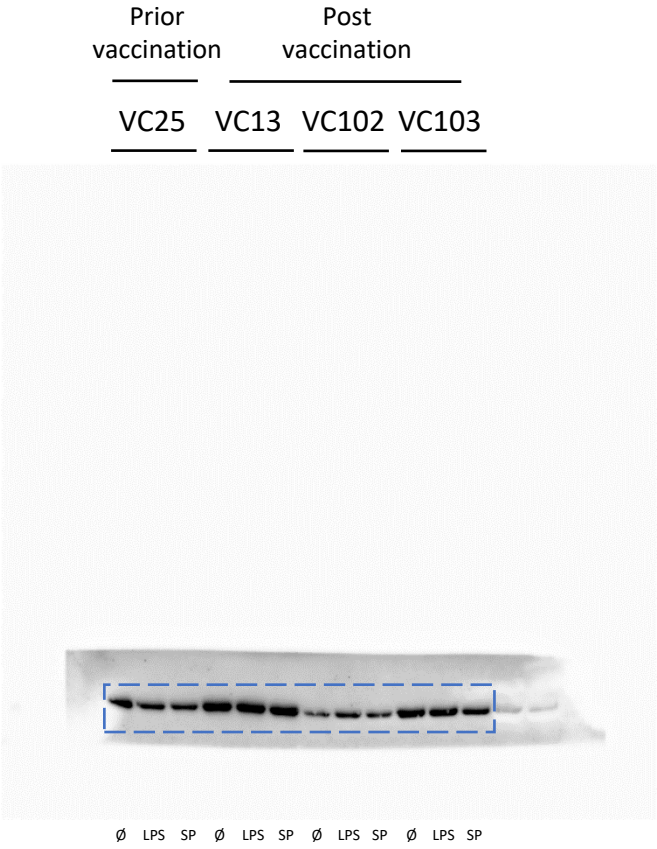

NLRP3

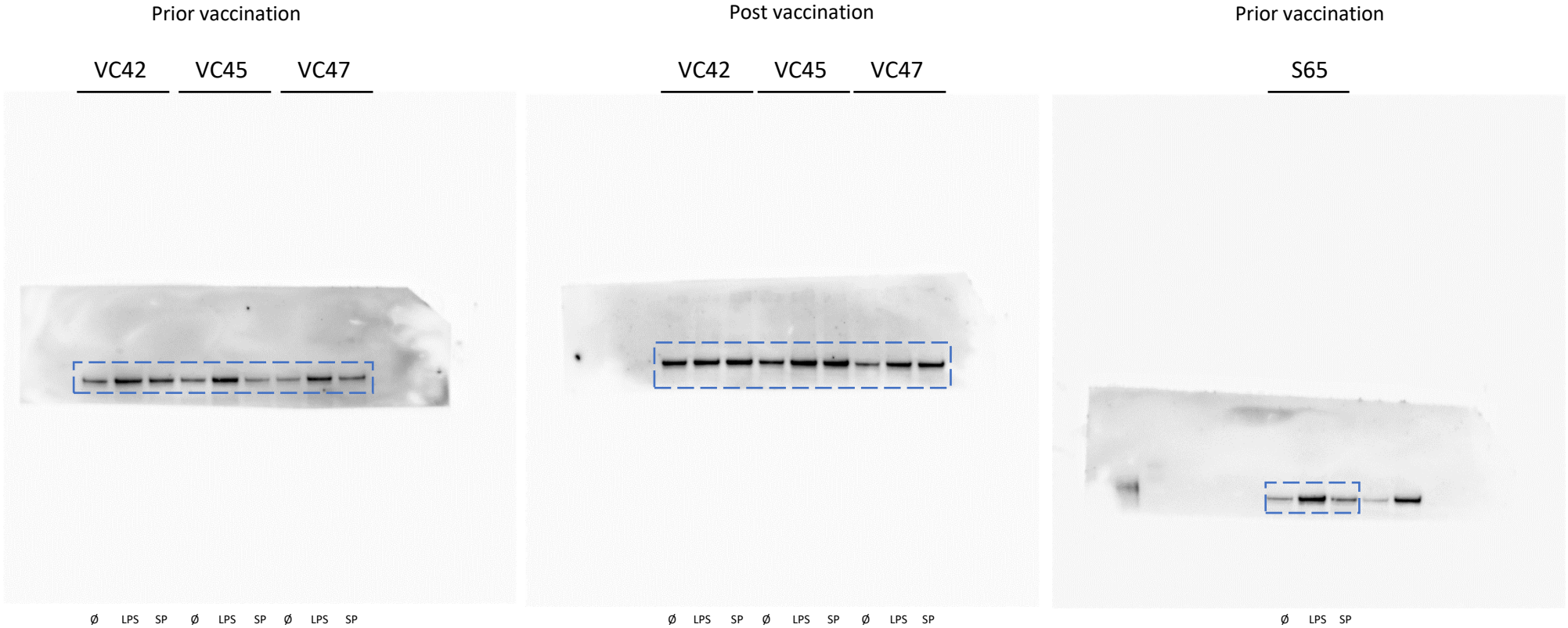

NLRP3

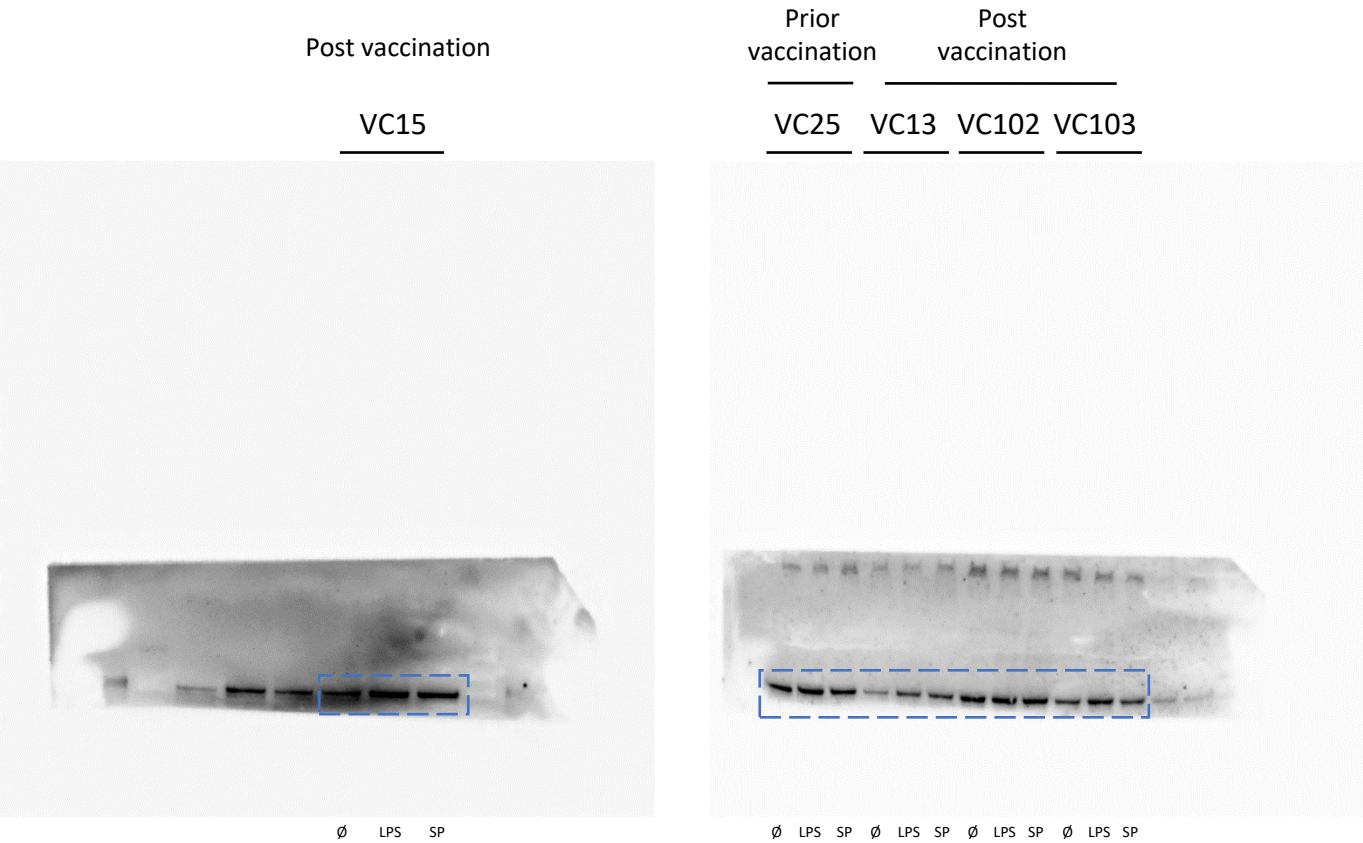

SOD2

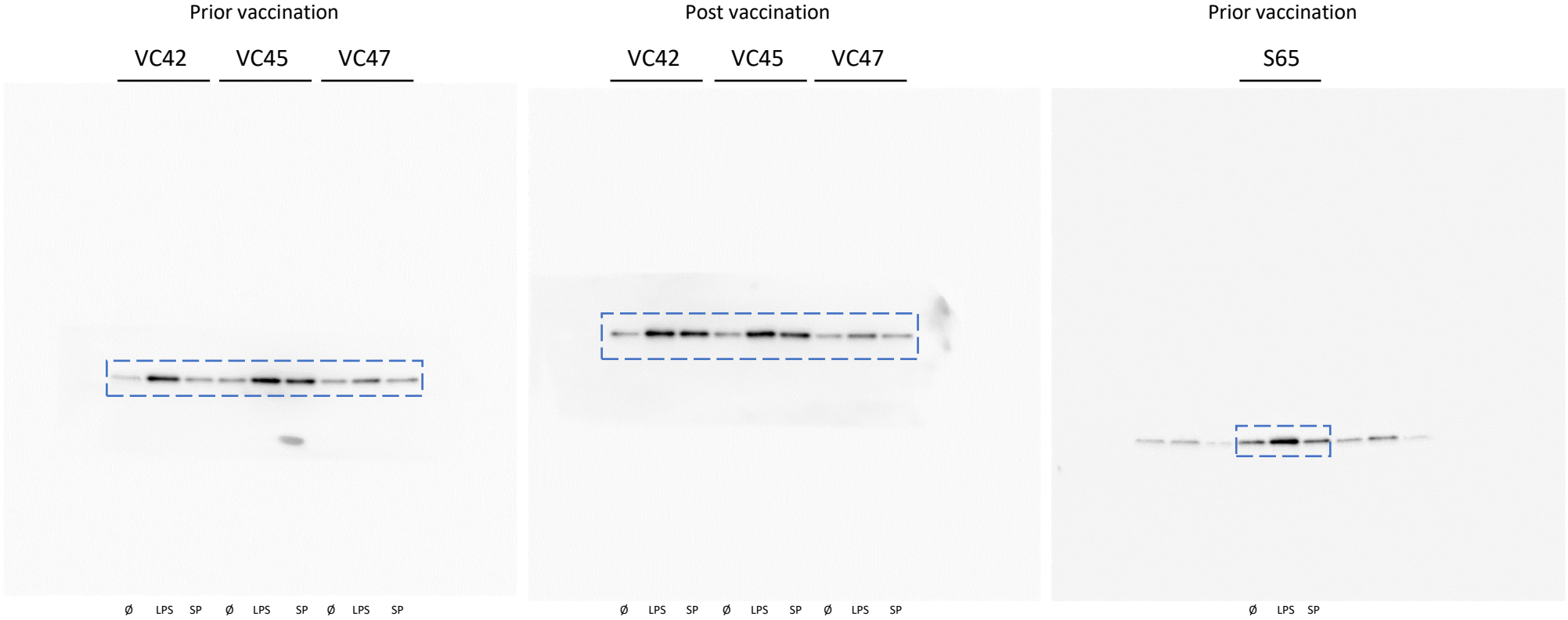

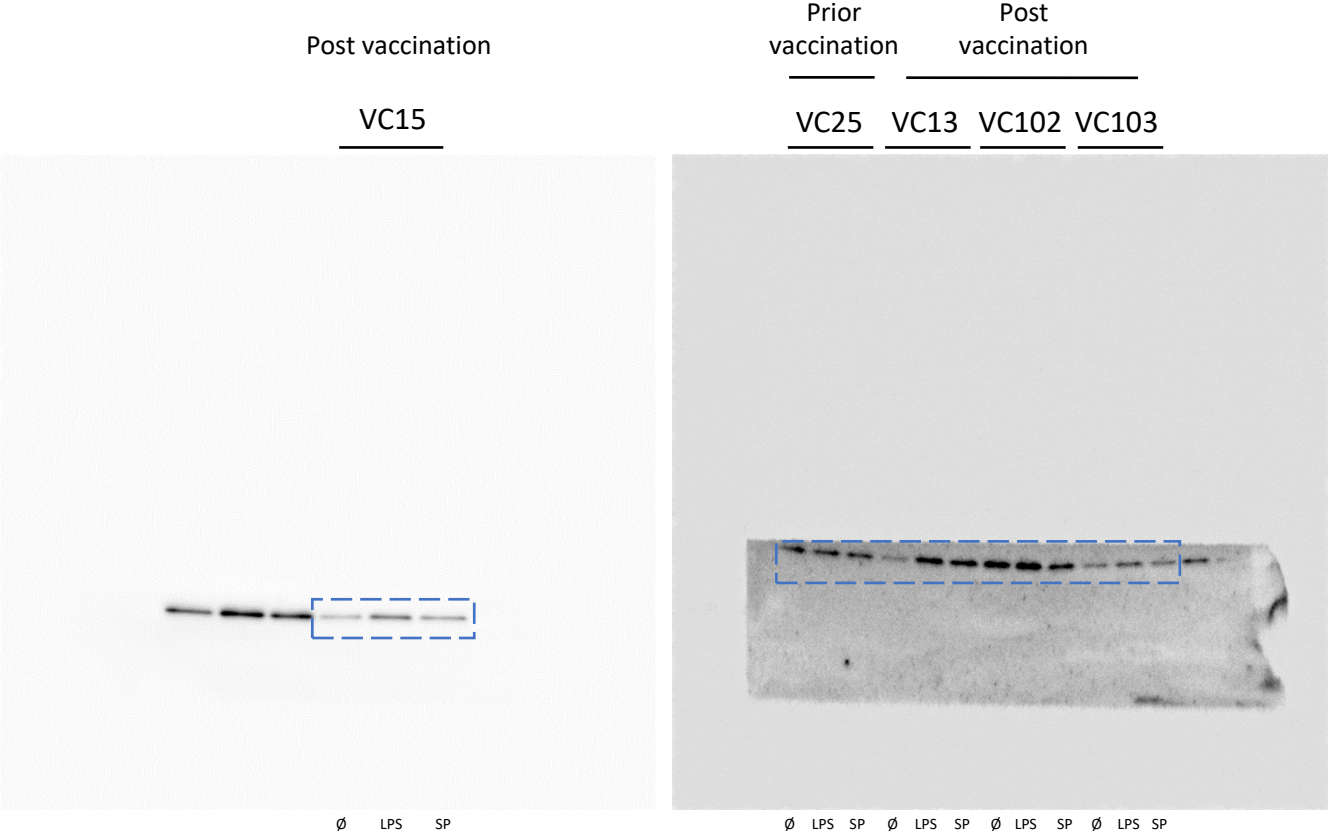

β-actin

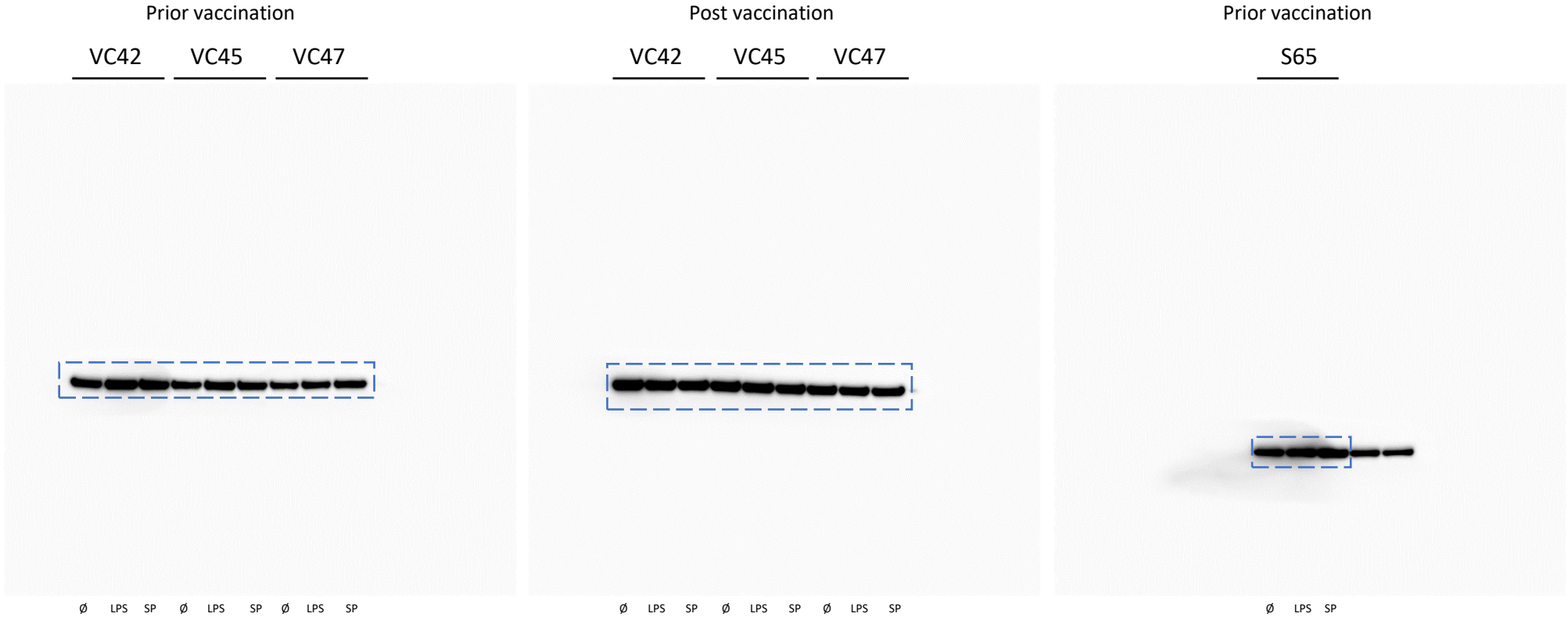

β-actin

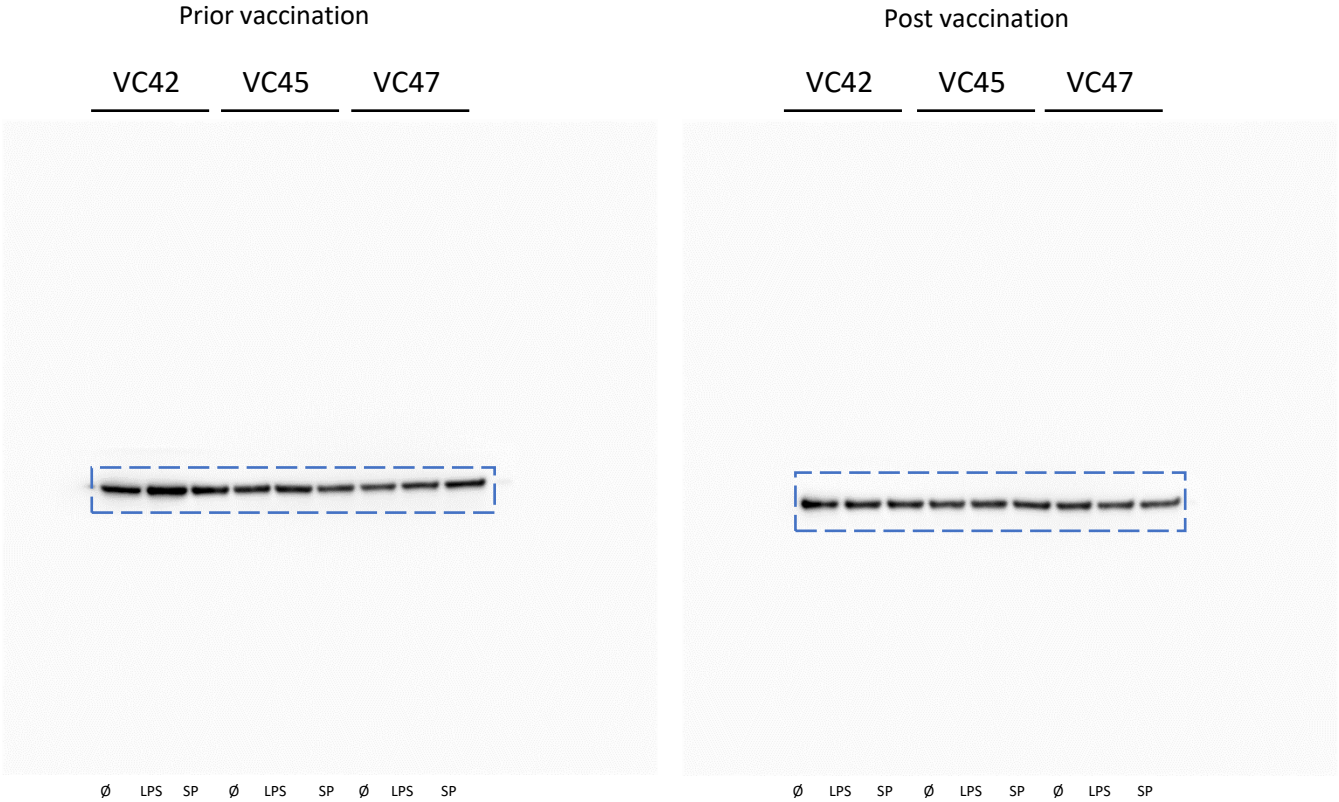

β-actin

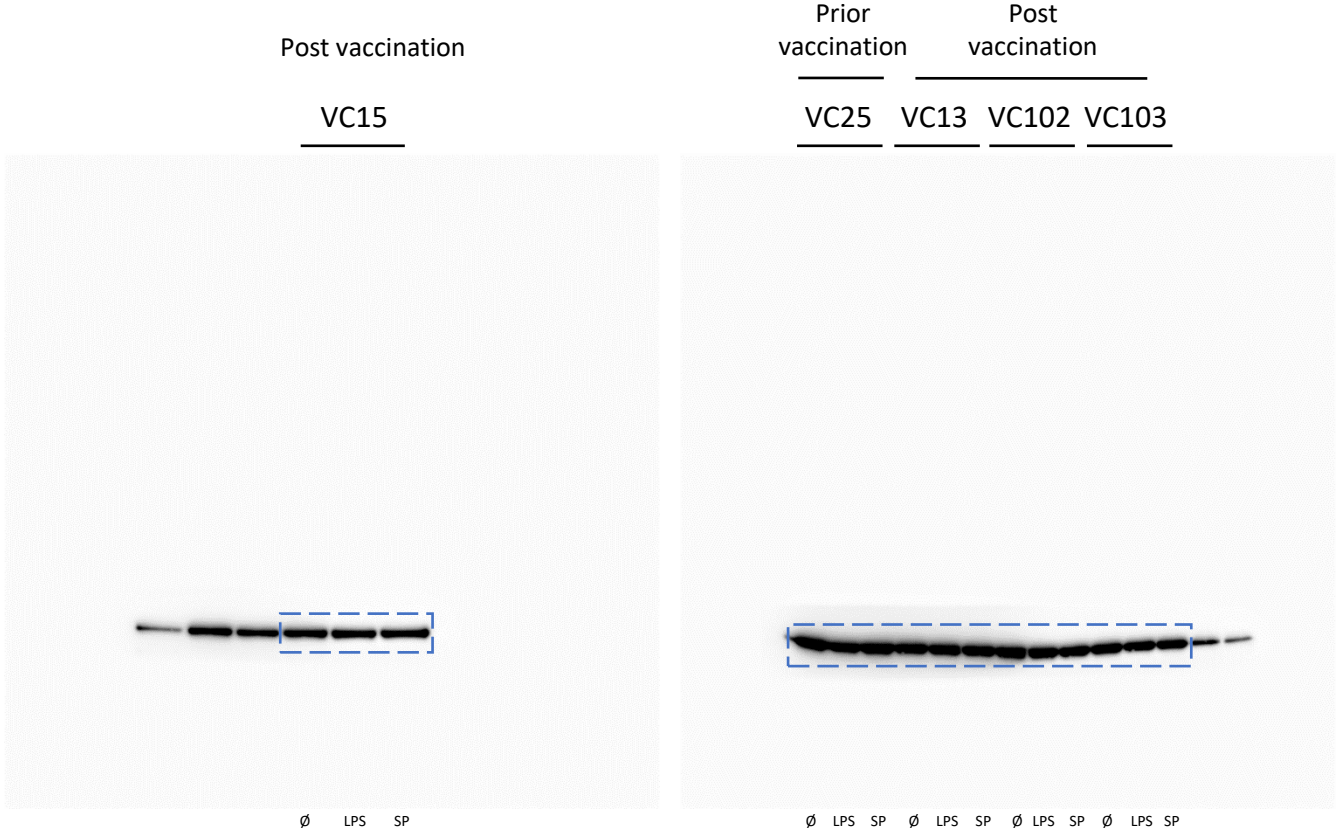

Supplement: Supplementary file 4 — Source Data for Figure 3 [file EMMM-14-e15888-s004.zip › Source data Fig3.pdf]
